# Opipramol dihydro­chloride

**DOI:** 10.1107/S1600536811042280

**Published:** 2011-10-22

**Authors:** Richard Betz, Thomas Gerber, Eric Hosten, Budanoor P. Siddaraju, Hemmige S. Yathirajan

**Affiliations:** a Nelson Mandela Metropolitan University, Summerstrand Campus, Department of Chemistry, University Way, Summerstrand, PO Box 77000, Port Elizabeth 6031, South Africa; b University of Mysore, Department of Studies in Chemistry, Manasagangotri, Mysore 570 006, India

## Abstract

The title compound (systematic name: 4-{3-[2-aza­tricyclo­[9.4.0.0^3,8^]penta­deca-1(15),3,5,7,11,13-hexaen-2-yl]prop­yl}-1-(2-hy­droxy­eth­yl)piperazine-1,4-diium dichloride), C_23_H_31_N_3_O^+^·2Cl^−^, is the dihydro­chloride of a piperazine derivative bearing a bulky 3-(5*H*-dibenz[*b*,*f*]azepin-5-yl)propyl substituent. Protonation took place on both N atoms of the piperazine unit. The diaza­cyclo­hexane ring adopts a chair conformation. N—H⋯Cl, O—H⋯Cl and C—H⋯Cl hydrogen bonding as well as C—H⋯O contacts connect the components into a three-dimensional network in the crystal. Two C—H⋯π contacts are also observed.

## Related literature

For applications of opipramol, see: Moller *et al.* (2001 ▶). For related structures, see: Jasinski *et al.* (2010 ▶); Fun *et al.* (2011 ▶); Siddegowda, Butcher *et al.* (2011 ▶); Siddegowda, Jasinski *et al.* (2011 ▶); Swamy *et al.* (2007 ▶). For graph-set analysis of hydrogen bonds, see: Etter *et al.* (1990 ▶); Bernstein *et al.* (1995 ▶). For puckering analysis, see: Cremer & Pople (1975 ▶); Boessenkool & Boeyens (1980 ▶).
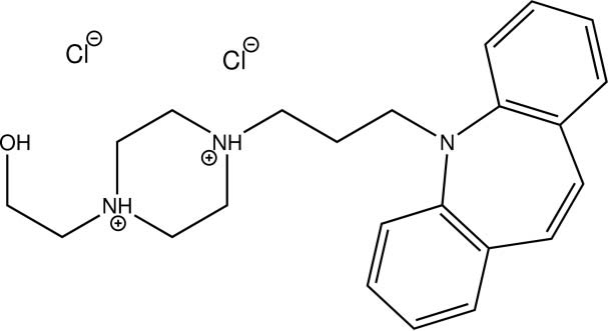


## Experimental

### 

#### Crystal data

C_23_H_31_N_3_O^2+^·2Cl^−^
*M* *_r_* = 436.41
Orthorhombic, 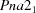

*a* = 33.6581 (6) Å
*b* = 9.4265 (2) Å
*c* = 6.8978 (1) Å
*V* = 2188.52 (7) Å^3^
*Z* = 4
Mo *K*α radiation
μ = 0.32 mm^−1^
*T* = 200 K
0.51 × 0.27 × 0.14 mm

#### Data collection

Bruker APEXII CCD diffractometer
20068 measured reflections
5253 independent reflections
4854 reflections with *I* > 2σ(*I*)
*R*_int_ = 0.030

#### Refinement

*R*[*F* ^2^ > 2σ(*F* ^2^)] = 0.027
*wR*(*F* ^2^) = 0.071
*S* = 1.03
5253 reflections
274 parameters
1 restraint
H atoms treated by a mixture of independent and constrained refinement
Δρ_max_ = 0.24 e Å^−3^
Δρ_min_ = −0.21 e Å^−3^
Absolute structure: Flack (1983 ▶), 2293 Friedel pairs
Flack parameter: −0.004 (33)

### 

Data collection: *APEX2* (Bruker, 2010 ▶); cell refinement: *SAINT* (Bruker, 2010 ▶); data reduction: *SAINT*; program(s) used to solve structure: *SHELXS97* (Sheldrick, 2008 ▶); program(s) used to refine structure: *SHELXL97* (Sheldrick, 2008 ▶); molecular graphics: *ORTEP-3* (Farrugia, 1997 ▶) and *Mercury* (Macrae *et al.*, 2008 ▶); software used to prepare material for publication: *SHELXL97* and *PLATON* (Spek, 2009 ▶).

## Supplementary Material

Crystal structure: contains datablock(s) I, global. DOI: 10.1107/S1600536811042280/mw2030sup1.cif

Supplementary material file. DOI: 10.1107/S1600536811042280/mw2030Isup2.cdx

Structure factors: contains datablock(s) I. DOI: 10.1107/S1600536811042280/mw2030Isup3.hkl

Supplementary material file. DOI: 10.1107/S1600536811042280/mw2030Isup4.cml

Additional supplementary materials:  crystallographic information; 3D view; checkCIF report

## Figures and Tables

**Table 1:** Hydrogen-bond geometry (Å, °) *Cg*2 is the centroid of the C11–C16 ring.

*D*—H⋯*A*	*D*—H	H⋯*A*	*D*⋯*A*	*D*—H⋯*A*
O1—H81⋯Cl1^i^	0.79 (2)	2.39 (2)	3.1701 (14)	169 (2)
N2—H72⋯Cl1	0.82 (2)	2.212 (19)	3.0057 (13)	163.0 (15)
N3—H73⋯Cl2^ii^	0.98 (3)	2.03 (3)	2.9972 (12)	171 (2)
C5—H5*A*⋯Cl1^iii^	0.99	2.82	3.7065 (15)	149
C5—H5*A*⋯O1^ii^	0.99	2.59	3.3373 (18)	133
C14—H14⋯Cl1^iv^	0.95	2.83	3.7233 (14)	157
C31—H31*A*⋯O1^ii^	0.99	2.54	3.2446 (18)	128
C31—H31*B*⋯Cl2^v^	0.99	2.75	3.5518 (14)	139
C32—H32*B*⋯Cl1^ii^	0.99	2.85	3.8246 (13)	169
C32—H32*A*⋯Cl2^vi^	0.99	2.85	3.7213 (16)	148
C6—H6*B*⋯Cl2^vi^	0.99	2.76	3.6634 (18)	152
C34—H34*A*⋯Cl2	0.99	2.82	3.5471 (13)	131
C16—H16⋯*Cg*2^iv^	0.95	2.98	3.6402 (16)	128
C23—H23⋯*Cg*2^vii^	0.95	2.67	3.4805 (18)	143

Symmetry codes: (i) 
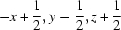
; (ii) 
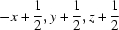
; (iii) 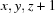; (iv) 
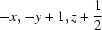
; (v) 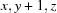; (vi) 
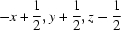
; (vii) 
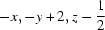
.

**Fig. 1.**
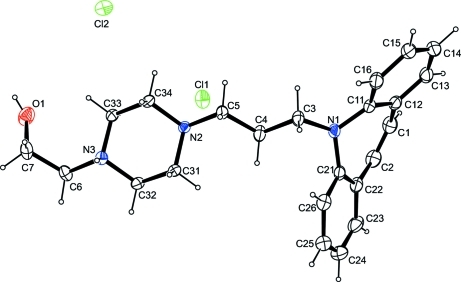
The molecular structure of the title compound, with atom labels and anisotropic displacement ellipsoids (drawn at 50% probability level).

**Fig. 2.**
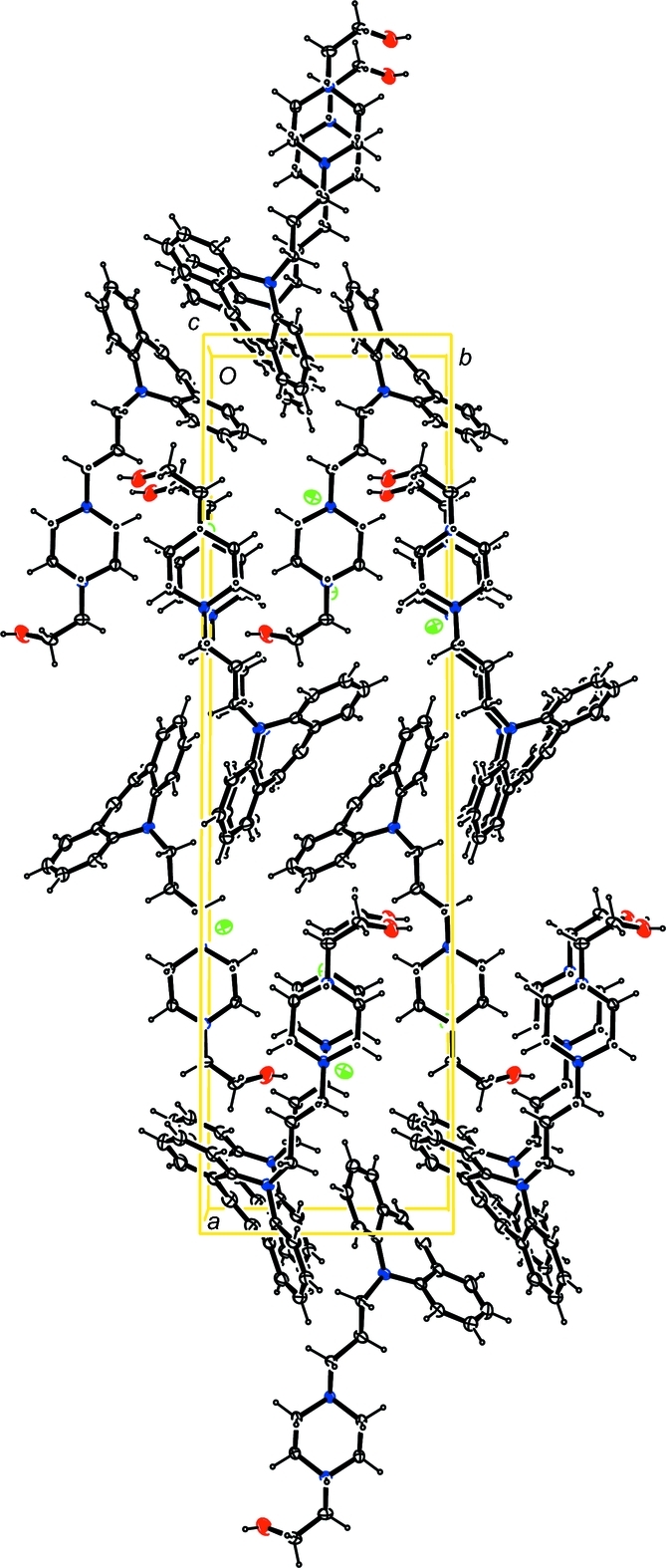
Molecular packing of the title compound, viewed along [0 0 - 1] (anisotropic displacement ellipsoids drawn at 50% probability level).

## supplementary crystallographic information

### Comment

Opipramol (systematic IUPAC name: 4-(3-{2-azatricyclo[9.4.0.0^3,8^]pentadeca- 1(15),3,5,7,11,13-hexaen-2-yl}propyl)-1-(2-hydroxyethyl)piperazin-1,4-diium dichloride is an antidepressant and anxiolytic typically used in the treatment of generalized anxiety disorder (Moller *et al.*, 2001). Opipramol is a tricyclic compound with no reuptake-inhibiting properties. However, it has pronounced D2-, 5-HT2-, and H1-blocking potential and high affinity to sigma receptors (sigma-1 and sigma-2). The crystal structures of opipramol dipicrate (Jasinski *et al.*, 2010), opipramol (Fun *et al.*, 2011), opipramolium fumarate (Siddegowda, Butcher *et al.*, 2011) and flupentixol dihydrochloride (Siddegowda, Jasinski *et al.*, 2011) have been reported recently. In view of the importance of the title compound we herein report its molecular and crystal structure.

Protonation took place exclusively on both nitrogen atoms of the piperazine unit. The diazacyclohexane ring adopts a ^4^*C*_1_ (^N3^*C*_N2_) conformation (Cremer & Pople, 1975). The more rigid skeleton of the seven-membered ring adopts a conformation in between a TC2 and a C6 conformation (*Q*_2_: 0.6128 (14) Å, *Q*_3_: 0.2043 (14) Å, π_2_: 177.14 (14)°, π_3_: 178.9 (4)°) (Boessenkool & Boeyens, 1980). The nitrogen atom sticks out of the least-squares plane defined by its atoms by 0.451 (1) Å (Fig. 1).

Apart from classical hydrogen bonds of the N–H···Cl and the O–H···Cl type, the crystal structure features a multitude of C–H···Cl contacts and two C–H···O contacts whose ranges invariably fall at least 0.1 Å below the sum of van-der-Waals radii of the respective atoms participating. The C–H···Cl contacts involve hydrogen atoms of methylene groups – both intracyclic as well as those on the hydroxyethyl side-chain – as well as one hydrogen atom on a phenyl ring. The C–H···O contacts all involve hydrogen atoms of the methylene groups located in the piperazine moiety and in the hydrocarbon chain connecting this to the remainder of the molecule. Both chloride anions attain pentacoordination *via* their combined contacts with hydrogen atoms, however, one of the anions features four instead of three C–H-supported contacts. In terms of graph-set analysis (Etter *et al.*, 1990; Bernstein *et al.*, 1995), the descriptor for the classical hydrogen bonds is *DDD* on the unitary level while the C–H···O contacts require a *C*^1^_1_(7)*C*^1^_1_(9) descriptor on the same level. A *DDDDDDD* descriptor on the unitary level is necessary to capture the C–H···Cl contacts. Furthermore, two C–H···*C*_g_ contacts can be observed involving hydrogen atoms on the phenyl rings as donors and the phenyl moiety comprised of the carbon atoms C11–C16. All of these interactions serve to connect the cations and anions into a three-dimensional network in the crystal. The shortest intercentroid distance between two aromatic systems was measured at 4.7319 (9) Å and is apparent between two different phenyl moieties.

The packing of the title compound in the crystal is shown in Figure 2.

### Experimental

The title compound was obtained as a gift sample from *R. L*. Fine Chem. Ltd., Bangalore, India. The compound was recrystallized from a 1:1 mixture of butan-1-one and benzene.

### Refinement

Carbon-bound H atoms were placed in calculated positions (C—H 0.95 Å for aromatic and vinylic hydrogen atoms, C—H 0.99 Å for methylene groups) and were included in the refinement in the riding model approximation, with *U*(H) set to 1.2*U*_eq_(C). The oxygen-bound H atom as well as both nitrogen-bound H atoms were located on a difference Fourier map and refined freely.

### Crystal data

**Table:**

C_23_H_31_N_3_O^2+^·2Cl^−^	*D*_x_ = 1.324 Mg m^−^^3^
*M**_r_* = 436.41	Melting point = 482–484 K
Orthorhombic, *P**n**a*2_1_	Mo *K*α radiation, λ = 0.71073 Å
Hall symbol: P 2c -2n	Cell parameters from 9954 reflections
*a* = 33.6581 (6) Å	θ = 2.4–28.3°
*b* = 9.4265 (2) Å	µ = 0.32 mm^−^^1^
*c* = 6.8978 (1) Å	*T* = 200 K
*V* = 2188.52 (7) Å^3^	Platelet, green
*Z* = 4	0.51 × 0.27 × 0.14 mm
*F*(000) = 928	

### Data collection

**Table:**

Bruker APEXII CCD diffractometer	4854 reflections with *I* > 2σ(*I*)
Radiation source: fine-focus sealed tube	*R*_int_ = 0.030
graphite	θ_max_ = 28.3°, θ_min_ = 2.2°
φ and ω scans	*h* = −44→44
20068 measured reflections	*k* = −12→9
5253 independent reflections	*l* = −9→9

### Refinement

**Table:**

Refinement on *F*^2^	Secondary atom site location: difference Fourier map
Least-squares matrix: full	Hydrogen site location: inferred from neighbouring sites
*R*[*F*^2^ > 2σ(*F*^2^)] = 0.027	H atoms treated by a mixture of independent and constrained refinement
*wR*(*F*^2^) = 0.071	*w* = 1/[σ^2^(*F*_o_^2^) + (0.0469*P*)^2^ + 0.0541*P*] where *P* = (*F*_o_^2^ + 2*F*_c_^2^)/3
*S* = 1.03	(Δ/σ)_max_ = 0.001
5253 reflections	Δρ_max_ = 0.24 e Å^−^^3^
274 parameters	Δρ_min_ = −0.21 e Å^−^^3^
1 restraint	Absolute structure: Flack (1983), 2293 Friedel pairs
Primary atom site location: structure-invariant direct methods	Flack parameter: −0.004 (33)

### Fractional atomic coordinates and isotropic or equivalent isotropic displacement parameters (Å^2^)

**Table:**

	*x*	*y*	*z*	*U*_iso_*/*U*_eq_	
O1	0.33522 (3)	0.24837 (14)	0.58052 (17)	0.0359 (3)	
H81	0.3356 (6)	0.173 (2)	0.631 (4)	0.044 (6)*	
N1	0.05595 (3)	0.73088 (11)	0.60732 (17)	0.0210 (2)	
N2	0.19011 (3)	0.51107 (11)	0.64833 (18)	0.0161 (2)	
H72	0.1886 (5)	0.5036 (16)	0.530 (3)	0.020 (4)*	
N3	0.27628 (3)	0.49616 (11)	0.69361 (16)	0.0174 (2)	
H73	0.2750 (7)	0.500 (2)	0.835 (5)	0.074 (8)*	
C1	0.00179 (4)	0.79746 (17)	0.2855 (2)	0.0294 (3)	
H1	−0.0128	0.7742	0.1721	0.035*	
C2	0.03133 (4)	0.89067 (17)	0.2622 (2)	0.0310 (3)	
H2	0.0356	0.9237	0.1337	0.037*	
C3	0.08286 (3)	0.64016 (15)	0.7199 (2)	0.0213 (3)	
H3A	0.0719	0.5428	0.7253	0.026*	
H3B	0.0846	0.6765	0.8543	0.026*	
C4	0.12436 (4)	0.63572 (14)	0.6312 (2)	0.0239 (3)	
H4A	0.1224	0.6198	0.4896	0.029*	
H4B	0.1379	0.7275	0.6529	0.029*	
C5	0.14816 (3)	0.51695 (13)	0.7235 (2)	0.0186 (2)	
H5A	0.1487	0.5309	0.8657	0.022*	
H5B	0.1349	0.4253	0.6970	0.022*	
C6	0.31889 (4)	0.49360 (15)	0.6266 (3)	0.0237 (3)	
H6A	0.3322	0.5816	0.6702	0.028*	
H6B	0.3195	0.4921	0.4832	0.028*	
C7	0.34174 (4)	0.36661 (17)	0.7029 (2)	0.0285 (3)	
H7A	0.3705	0.3890	0.7074	0.034*	
H7B	0.3328	0.3441	0.8362	0.034*	
C11	0.01455 (3)	0.69862 (13)	0.6216 (2)	0.0207 (2)	
C12	−0.01079 (4)	0.72710 (15)	0.4633 (2)	0.0232 (3)	
C13	−0.05001 (4)	0.67850 (16)	0.4742 (2)	0.0292 (3)	
H13	−0.0675	0.6980	0.3694	0.035*	
C14	−0.06431 (4)	0.60348 (16)	0.6307 (3)	0.0332 (3)	
H14	−0.0910	0.5707	0.6328	0.040*	
C15	−0.03922 (4)	0.57682 (16)	0.7842 (3)	0.0332 (4)	
H15	−0.0485	0.5242	0.8924	0.040*	
C16	−0.00025 (4)	0.62655 (16)	0.7816 (2)	0.0272 (3)	
H16	0.0164	0.6110	0.8908	0.033*	
C21	0.06763 (4)	0.87575 (14)	0.5829 (2)	0.0219 (3)	
C22	0.05776 (4)	0.94775 (16)	0.4107 (2)	0.0250 (3)	
C23	0.07445 (5)	1.08179 (16)	0.3787 (3)	0.0338 (4)	
H23	0.0679	1.1315	0.2633	0.041*	
C24	0.10005 (5)	1.14375 (17)	0.5089 (3)	0.0400 (4)	
H24	0.1118	1.2331	0.4808	0.048*	
C25	0.10856 (5)	1.07525 (18)	0.6806 (3)	0.0383 (4)	
H25	0.1256	1.1186	0.7728	0.046*	
C26	0.09204 (4)	0.94189 (17)	0.7189 (3)	0.0294 (3)	
H26	0.0975	0.8961	0.8386	0.035*	
C31	0.21421 (3)	0.63744 (14)	0.7062 (2)	0.0199 (3)	
H31A	0.2154	0.6432	0.8494	0.024*	
H31B	0.2012	0.7248	0.6578	0.024*	
C32	0.25605 (3)	0.62810 (13)	0.6249 (2)	0.0199 (2)	
H32A	0.2549	0.6281	0.4815	0.024*	
H32B	0.2715	0.7121	0.6666	0.024*	
C33	0.25223 (3)	0.37093 (13)	0.6327 (2)	0.0190 (2)	
H33A	0.2653	0.2829	0.6782	0.023*	
H33B	0.2508	0.3674	0.4895	0.023*	
C34	0.21058 (4)	0.37928 (14)	0.7157 (2)	0.0191 (2)	
H34A	0.1952	0.2952	0.6742	0.023*	
H34B	0.2119	0.3790	0.8591	0.023*	
Cl1	0.172378 (10)	0.43277 (4)	0.23488 (5)	0.03102 (9)	
Cl2	0.217571 (10)	0.00951 (3)	0.62670 (6)	0.02579 (8)	

### Atomic displacement parameters (Å^2^)

**Table:**

	*U*^11^	*U*^22^	*U*^33^	*U*^12^	*U*^13^	*U*^23^
O1	0.0419 (6)	0.0321 (6)	0.0337 (7)	0.0108 (5)	−0.0089 (5)	−0.0078 (5)
N1	0.0168 (4)	0.0210 (5)	0.0252 (6)	0.0029 (4)	−0.0012 (4)	0.0058 (5)
N2	0.0176 (5)	0.0147 (5)	0.0161 (6)	0.0006 (4)	−0.0018 (4)	0.0003 (4)
N3	0.0177 (5)	0.0200 (6)	0.0146 (5)	0.0015 (4)	−0.0007 (4)	−0.0010 (4)
C1	0.0297 (7)	0.0346 (8)	0.0240 (7)	0.0056 (6)	−0.0057 (5)	−0.0003 (6)
C2	0.0354 (7)	0.0339 (8)	0.0237 (8)	0.0069 (6)	0.0000 (6)	0.0072 (6)
C3	0.0179 (5)	0.0240 (6)	0.0219 (6)	0.0043 (5)	0.0001 (5)	0.0045 (6)
C4	0.0197 (6)	0.0267 (7)	0.0253 (7)	0.0060 (5)	0.0020 (5)	0.0062 (6)
C5	0.0150 (5)	0.0198 (6)	0.0209 (6)	0.0013 (4)	−0.0003 (5)	0.0007 (6)
C6	0.0164 (5)	0.0307 (7)	0.0241 (6)	−0.0005 (5)	0.0006 (6)	−0.0026 (6)
C7	0.0212 (6)	0.0377 (8)	0.0268 (7)	0.0080 (6)	−0.0065 (5)	−0.0059 (6)
C11	0.0181 (5)	0.0179 (6)	0.0262 (6)	0.0036 (4)	0.0004 (5)	−0.0018 (6)
C12	0.0224 (6)	0.0200 (7)	0.0272 (7)	0.0040 (5)	−0.0030 (5)	−0.0045 (6)
C13	0.0223 (7)	0.0274 (7)	0.0378 (8)	0.0037 (6)	−0.0064 (6)	−0.0095 (7)
C14	0.0221 (6)	0.0259 (7)	0.0517 (10)	−0.0012 (5)	0.0044 (7)	−0.0092 (8)
C15	0.0259 (7)	0.0245 (7)	0.0492 (11)	0.0017 (6)	0.0117 (6)	0.0049 (7)
C16	0.0229 (6)	0.0276 (7)	0.0312 (8)	0.0053 (6)	0.0038 (5)	0.0044 (6)
C21	0.0166 (6)	0.0210 (6)	0.0283 (8)	0.0044 (5)	0.0031 (5)	0.0019 (6)
C22	0.0228 (6)	0.0242 (7)	0.0280 (7)	0.0052 (6)	0.0045 (6)	0.0045 (6)
C23	0.0374 (8)	0.0238 (8)	0.0403 (9)	0.0067 (6)	0.0072 (7)	0.0092 (7)
C24	0.0353 (8)	0.0211 (8)	0.0637 (12)	−0.0021 (7)	0.0059 (8)	0.0027 (8)
C25	0.0284 (7)	0.0265 (8)	0.0598 (13)	0.0009 (6)	−0.0061 (7)	−0.0085 (7)
C26	0.0258 (6)	0.0265 (7)	0.0359 (8)	0.0040 (6)	−0.0024 (6)	0.0002 (7)
C31	0.0190 (5)	0.0152 (6)	0.0256 (7)	0.0010 (5)	−0.0004 (5)	−0.0032 (5)
C32	0.0218 (6)	0.0159 (5)	0.0220 (6)	−0.0002 (4)	−0.0004 (5)	0.0013 (6)
C33	0.0190 (5)	0.0161 (5)	0.0218 (6)	0.0023 (4)	−0.0001 (5)	−0.0019 (6)
C34	0.0204 (5)	0.0146 (6)	0.0224 (6)	0.0019 (5)	−0.0002 (5)	0.0026 (6)
Cl1	0.02975 (16)	0.0431 (2)	0.02020 (15)	0.00569 (15)	−0.00392 (14)	−0.00588 (17)
Cl2	0.03646 (17)	0.02445 (16)	0.01645 (14)	−0.00133 (13)	0.00162 (14)	−0.00090 (14)

### Geometric parameters (Å, °)

**Table:**

O1—C7	1.4155 (19)	C11—C16	1.388 (2)
O1—H81	0.79 (2)	C11—C12	1.4112 (19)
N1—C11	1.4296 (15)	C12—C13	1.3995 (19)
N1—C21	1.4311 (17)	C13—C14	1.377 (2)
N1—C3	1.4681 (16)	C13—H13	0.9500
N2—C34	1.4947 (16)	C14—C15	1.377 (2)
N2—C31	1.4955 (16)	C14—H14	0.9500
N2—C5	1.5049 (16)	C15—C16	1.393 (2)
N2—H72	0.82 (2)	C15—H15	0.9500
N3—C33	1.4918 (16)	C16—H16	0.9500
N3—C32	1.4950 (16)	C21—C26	1.394 (2)
N3—C6	1.5069 (16)	C21—C22	1.408 (2)
N3—H73	0.98 (3)	C22—C23	1.400 (2)
C1—C2	1.336 (2)	C23—C24	1.375 (3)
C1—C12	1.457 (2)	C23—H23	0.9500
C1—H1	0.9500	C24—C25	1.379 (3)
C2—C22	1.460 (2)	C24—H24	0.9500
C2—H2	0.9500	C25—C26	1.400 (2)
C3—C4	1.5256 (17)	C25—H25	0.9500
C3—H3A	0.9900	C26—H26	0.9500
C3—H3B	0.9900	C31—C32	1.5183 (17)
C4—C5	1.5168 (18)	C31—H31A	0.9900
C4—H4A	0.9900	C31—H31B	0.9900
C4—H4B	0.9900	C32—H32A	0.9900
C5—H5A	0.9900	C32—H32B	0.9900
C5—H5B	0.9900	C33—C34	1.5160 (17)
C6—C7	1.517 (2)	C33—H33A	0.9900
C6—H6A	0.9900	C33—H33B	0.9900
C6—H6B	0.9900	C34—H34A	0.9900
C7—H7A	0.9900	C34—H34B	0.9900
C7—H7B	0.9900		
			
C7—O1—H81	116.3 (18)	C13—C12—C1	117.91 (13)
C11—N1—C21	118.62 (10)	C11—C12—C1	124.22 (13)
C11—N1—C3	116.17 (10)	C14—C13—C12	122.66 (14)
C21—N1—C3	116.65 (10)	C14—C13—H13	118.7
C34—N2—C31	109.20 (10)	C12—C13—H13	118.7
C34—N2—C5	110.86 (10)	C15—C14—C13	118.80 (13)
C31—N2—C5	112.80 (10)	C15—C14—H14	120.6
C34—N2—H72	105.4 (11)	C13—C14—H14	120.6
C31—N2—H72	111.5 (11)	C14—C15—C16	120.43 (15)
C5—N2—H72	106.8 (11)	C14—C15—H15	119.8
C33—N3—C32	108.78 (10)	C16—C15—H15	119.8
C33—N3—C6	114.68 (10)	C11—C16—C15	120.82 (14)
C32—N3—C6	110.47 (10)	C11—C16—H16	119.6
C33—N3—H73	106.9 (13)	C15—C16—H16	119.6
C32—N3—H73	105.3 (13)	C26—C21—C22	119.42 (13)
C6—N3—H73	110.3 (13)	C26—C21—N1	120.63 (12)
C2—C1—C12	128.07 (14)	C22—C21—N1	119.64 (13)
C2—C1—H1	116.0	C23—C22—C21	118.25 (14)
C12—C1—H1	116.0	C23—C22—C2	117.80 (14)
C1—C2—C22	127.67 (14)	C21—C22—C2	123.94 (14)
C1—C2—H2	116.2	C24—C23—C22	122.13 (15)
C22—C2—H2	116.2	C24—C23—H23	118.9
N1—C3—C4	111.63 (11)	C22—C23—H23	118.9
N1—C3—H3A	109.3	C23—C24—C25	119.48 (15)
C4—C3—H3A	109.3	C23—C24—H24	120.3
N1—C3—H3B	109.3	C25—C24—H24	120.3
C4—C3—H3B	109.3	C24—C25—C26	120.03 (16)
H3A—C3—H3B	108.0	C24—C25—H25	120.0
C5—C4—C3	109.60 (11)	C26—C25—H25	120.0
C5—C4—H4A	109.8	C21—C26—C25	120.56 (16)
C3—C4—H4A	109.8	C21—C26—H26	119.7
C5—C4—H4B	109.8	C25—C26—H26	119.7
C3—C4—H4B	109.8	N2—C31—C32	111.00 (11)
H4A—C4—H4B	108.2	N2—C31—H31A	109.4
N2—C5—C4	112.22 (11)	C32—C31—H31A	109.4
N2—C5—H5A	109.2	N2—C31—H31B	109.4
C4—C5—H5A	109.2	C32—C31—H31B	109.4
N2—C5—H5B	109.2	H31A—C31—H31B	108.0
C4—C5—H5B	109.2	N3—C32—C31	110.71 (11)
H5A—C5—H5B	107.9	N3—C32—H32A	109.5
N3—C6—C7	112.88 (12)	C31—C32—H32A	109.5
N3—C6—H6A	109.0	N3—C32—H32B	109.5
C7—C6—H6A	109.0	C31—C32—H32B	109.5
N3—C6—H6B	109.0	H32A—C32—H32B	108.1
C7—C6—H6B	109.0	N3—C33—C34	110.76 (10)
H6A—C6—H6B	107.8	N3—C33—H33A	109.5
O1—C7—C6	109.62 (12)	C34—C33—H33A	109.5
O1—C7—H7A	109.7	N3—C33—H33B	109.5
C6—C7—H7A	109.7	C34—C33—H33B	109.5
O1—C7—H7B	109.7	H33A—C33—H33B	108.1
C6—C7—H7B	109.7	N2—C34—C33	110.62 (10)
H7A—C7—H7B	108.2	N2—C34—H34A	109.5
C16—C11—C12	119.44 (12)	C33—C34—H34A	109.5
C16—C11—N1	120.57 (12)	N2—C34—H34B	109.5
C12—C11—N1	119.67 (12)	C33—C34—H34B	109.5
C13—C12—C11	117.80 (13)	H34A—C34—H34B	108.1
			
C12—C1—C2—C22	−1.6 (3)	C11—N1—C21—C26	120.11 (14)
C11—N1—C3—C4	154.28 (12)	C3—N1—C21—C26	−26.61 (18)
C21—N1—C3—C4	−58.17 (16)	C11—N1—C21—C22	−66.34 (17)
N1—C3—C4—C5	−167.31 (11)	C3—N1—C21—C22	146.94 (12)
C34—N2—C5—C4	−170.35 (11)	C26—C21—C22—C23	2.8 (2)
C31—N2—C5—C4	66.82 (15)	N1—C21—C22—C23	−170.80 (12)
C3—C4—C5—N2	−177.44 (11)	C26—C21—C22—C2	−177.67 (13)
C33—N3—C6—C7	−60.37 (17)	N1—C21—C22—C2	8.7 (2)
C32—N3—C6—C7	176.29 (12)	C1—C2—C22—C23	−153.31 (16)
N3—C6—C7—O1	84.54 (16)	C1—C2—C22—C21	27.2 (2)
C21—N1—C11—C16	−123.35 (14)	C21—C22—C23—C24	0.3 (2)
C3—N1—C11—C16	23.53 (18)	C2—C22—C23—C24	−179.21 (15)
C21—N1—C11—C12	63.22 (17)	C22—C23—C24—C25	−2.6 (3)
C3—N1—C11—C12	−149.90 (12)	C23—C24—C25—C26	1.7 (3)
C16—C11—C12—C13	−1.05 (19)	C22—C21—C26—C25	−3.7 (2)
N1—C11—C12—C13	172.46 (13)	N1—C21—C26—C25	169.85 (13)
C16—C11—C12—C1	−177.82 (14)	C24—C25—C26—C21	1.4 (2)
N1—C11—C12—C1	−4.3 (2)	C34—N2—C31—C32	57.10 (15)
C2—C1—C12—C13	155.85 (16)	C5—N2—C31—C32	−179.15 (11)
C2—C1—C12—C11	−27.4 (2)	C33—N3—C32—C31	58.15 (15)
C11—C12—C13—C14	−0.8 (2)	C6—N3—C32—C31	−175.15 (12)
C1—C12—C13—C14	176.16 (14)	N2—C31—C32—N3	−58.39 (15)
C12—C13—C14—C15	1.0 (2)	C32—N3—C33—C34	−58.79 (15)
C13—C14—C15—C16	0.8 (2)	C6—N3—C33—C34	176.97 (12)
C12—C11—C16—C15	2.8 (2)	C31—N2—C34—C33	−57.48 (14)
N1—C11—C16—C15	−170.66 (13)	C5—N2—C34—C33	177.64 (11)
C14—C15—C16—C11	−2.7 (2)	N3—C33—C34—N2	59.46 (15)

### Hydrogen-bond geometry (Å, °)

**Table:**

Cg2 is the centroid of the C11–C16 ring.

**Table:**

*D*—H···*A*	*D*—H	H···*A*	*D*···*A*	*D*—H···*A*
O1—H81···Cl1^i^	0.79 (2)	2.39 (2)	3.1701 (14)	169 (2)
N2—H72···Cl1	0.82 (2)	2.212 (19)	3.0057 (13)	163.0 (15)
N3—H73···Cl2^ii^	0.98 (3)	2.03 (3)	2.9972 (12)	171 (2)
C5—H5A···Cl1^iii^	0.99	2.82	3.7065 (15)	149.
C5—H5A···O1^ii^	0.99	2.59	3.3373 (18)	133.
C14—H14···Cl1^iv^	0.95	2.83	3.7233 (14)	157.
C31—H31A···O1^ii^	0.99	2.54	3.2446 (18)	128.
C31—H31B···Cl2^v^	0.99	2.75	3.5518 (14)	139.
C32—H32B···Cl1^ii^	0.99	2.85	3.8246 (13)	169.
C32—H32A···Cl2^vi^	0.99	2.85	3.7213 (16)	148.
C6—H6B···Cl2^vi^	0.99	2.76	3.6634 (18)	152.
C34—H34A···Cl2	0.99	2.82	3.5471 (13)	131.
C16—H16···Cg2^iv^	0.95	2.98	3.6402 (16)	128
C23—H23···Cg2^vii^	0.95	2.67	3.4805 (18)	143

Symmetry codes: (i) −*x*+1/2, *y*−1/2, *z*+1/2; (ii) −*x*+1/2, *y*+1/2, *z*+1/2; (iii) *x*, *y*, *z*+1; (iv) −*x*, −*y*+1, *z*+1/2; (v) *x*, *y*+1, *z*; (vi) −*x*+1/2, *y*+1/2, *z*−1/2; (vii) −*x*, −*y*+2, *z*−1/2.

## References

[bb1] Bernstein, J., Davis, R. E., Shimoni, L. & Chang, N.-L. (1995). *Angew. Chem. Int. Ed. Engl.* **34**, 1555–1573.

[bb2] Boessenkool, I. K. & Boeyens, J. C. A. (1980). *J. Cryst. Mol. Struct.* **10**, 11–18.

[bb3] Bruker (2010). *APEX2* and *SAINT* Bruker AXS Inc., Madison, Wisconsin, USA.

[bb4] Cremer, D. & Pople, J. A. (1975). *J. Am. Chem. Soc.* **97**, 1354–1358.

[bb5] Etter, M. C., MacDonald, J. C. & Bernstein, J. (1990). *Acta Cryst.* B**46**, 256–262. 10.1107/s0108768189012929 2344397

[bb6] Farrugia, L. J. (1997). *J. Appl. Cryst.* **30**, 565.

[bb7] Flack, H. D. (1983). *Acta Cryst.* A**39**, 876–881.

[bb8] Fun, H.-K., Loh, W.-S., Siddegowda, M. S., Yathirajan, H. S. & Narayana, B. (2011). *Acta Cryst.* E**67**, o1598. 10.1107/S1600536811021131 PMC3151965 21837006

[bb9] Jasinski, J. P., Pek, A. E., Siddaraju, B. P., Yathirajan, H. S. & Narayana, B. (2010). *Acta Cryst.* E**66**, o1979–o1980. 10.1107/S1600536810026565 PMC3007231 21588296

[bb10] Macrae, C. F., Bruno, I. J., Chisholm, J. A., Edgington, P. R., McCabe, P., Pidcock, E., Rodriguez-Monge, L., Taylor, R., van de Streek, J. & Wood, P. A. (2008). *J. Appl. Cryst.* **41**, 466–470.

[bb11] Moller, H. J., Volz, H. P., Reimann, I. W. & Stoll, K. D. (2001). *J. Clin. Psychopharmacol.* **21**, 59–65. 10.1097/00004714-200102000-00011 11199949

[bb12] Sheldrick, G. M. (2008). *Acta Cryst.* A**64**, 112–122. 10.1107/S0108767307043930 18156677

[bb13] Siddegowda, M. S., Butcher, R. J., Akkurt, M., Yathirajan, H. S. & Narayana, B. (2011). *Acta Cryst.* E**67**, o2079–o2080. 10.1107/S1600536811028182 PMC3213522 22091101

[bb14] Siddegowda, M. S., Jasinski, J. P., Golen, J. A., Yathirajan, H. S. & Swamy, M. T. (2011). *Acta Cryst.* E**67**, o2296. 10.1107/S160053681103159X PMC3200775 22064675

[bb15] Spek, A. L. (2009). *Acta Cryst.* D**65**, 148–155. 10.1107/S090744490804362X PMC2631630 19171970

[bb16] Swamy, M. T., Ashok, M. A., Yathirajan, H. S., Narayana, B. & Bolte, M. (2007). *Acta Cryst.* E**63**, o4919.

